# A Personalized Approach in Progressive Multiple Sclerosis: The Current Status of Disease Modifying Therapies (DMTs) and Future Perspectives

**DOI:** 10.3390/ijms17101725

**Published:** 2016-10-17

**Authors:** Emanuele D’Amico, Francesco Patti, Aurora Zanghì, Mario Zappia

**Affiliations:** Multiple Sclerosis Center, Policlinico G. Rodolico, via Santa Sofia, 78 Catania 95123, Italy; emanueledamico82@gmail.com (E.D.); aurora.zanghi@yahoo.it (A.Z.); m.zappia@unict.it (M.Z.)

**Keywords:** personalized treatment, progressive multiple sclerosis, future perspectives

## Abstract

Using the term of progressive multiple sclerosis (PMS), we considered a combined population of persons with secondary progressive MS (SPMS) and primary progressive MS (PPMS). These forms of MS cannot be challenged with efficacy by the licensed therapy. In the last years, several measures of risk estimation were developed for predicting clinical course in MS, but none is specific for the PMS forms. Personalized medicine is a therapeutic approach, based on identifying what might be the best therapy for an individual patient, taking into account the risk profile. We need to achieve more accurate estimates of useful predictors in PMS, including unconventional and qualitative markers which are not yet currently available or practicable routine diagnostics. The evaluation of an individual patient is based on the profile of disease activity.Within the neurology field, PMS is one of the fastest-moving going into the future.

## 1. Introduction

Multiple sclerosis (MS) is a chronic autoimmune disease of the central nervous system (CNS), usually characterized by a relapsing-remitting (RR) course, or by disability accrual over an extreme variability of time. At diagnosis, about 75% of persons with MS suffer from RRMS, while about 25% of such persons will convert to secondary progressive MS (SPMS) within two to three decades from the onset [1]. About 10% of patients with MS manifest primary progressive MS (PPMS) at diagnosis, and they show gradual worsening of neurological disability from symptom onset [1]. With the term progressive MS (PMS), we usually refer to the combined population of secondary progressive MS (SPMS) and primary progressive MS (PPMS) that still remains a distinct phenotype in the 2013 revision of clinical course in MS [2].

The licensed MS therapies have shown ability in the management of RRMS forms, but they have not shown efficacy for PMS. For this reason, establishing effective therapies for PMS is highly recommended and it is one of the greatest priorities for the global MS community, because more than one million people suffer from MS [3]. Deeper interest in this challenging topic is evidenced by the establishment in 2012 of the Progressive MS Alliance, an international group of MS specialists, aiming to focus their efforts on the development of new therapy for PMS [4].

The approach of common clinical trials cannot be applied to PMS (due to the lack of treatment efficacy), and for this group of persons, the treatment decisions must be made on an individual basis [5]. The clinical management of PMS represents the paradigm of personalized treatment in MS. A personalized approach should be based on the identification of disease course, treatment response biomarkers, and on the evaluation of the risk profile for each therapy. Few biomarkers have made their way into clinical management of MS, so far.

We highlight the current status of a personalized approach in the clinical management of PMS, and the challenges of defining candidate predictors of clinical course and putative therapeutics.

## 2. Insights into Pathogenesis of Progressive Forms

The exact mechanisms of disease progression in PMS are still unknown, but a pathogenic role of B lymphocytes in some patients with early PPMS was suggested [6]. Studies on post-mortem brain tissue from SPMS patients showed meningeal lymphoid follicles containing proliferating B cells [7]. No such data have been reported in PPMS brain tissue. Here, diffuse T-cells and B-cell-driven meningeal inflammation has been shown, and it was associated with severe clinical course [8]. Furthermore, B-cell follicles were found in 41.4% of patients with SPMS, but not in patients with PPMS. The presence of meningeal follicles in SPMS patients was associated with a younger age at disease onset, high disability, and more pronounced demyelination, microglia activation, and axonal damage. These structures were usually adjacent to large cortical lesions, supporting the hypothesis that B cell follicles could exacerbate the effects of humoral immunity with major damage of cortical structures [7].

Recently, it was shown that a widespread demyelinating pathology (predominant in the cortical grey matter, with subpial localization) is associated to the inflammation of the overlying meninges. Therefore, inflammation accompanies neuronal pathology both in SPMS and PPMS patients, although in PPMS the pathological changes are less extensive when compared to SPMS. These data help to define the controversial role of immune response and inflammation in SPMS and PPMS, suggesting that immunotherapy will need to target this inflammation [8].

Another pathogenetic hypothesis regards the mitochondrial dysfunction and oxidative stress. 

A recent study suggested that MS progression is associated with low systemic oxidative activity accompanied with a dysregulated immune response and consequent CNS inflammation cyclooxygenase-dependent. Details were determined by liquid chromatography–tandem mass spectrometry non enzymatic (F2-isoprostanes) and enzymatic oxidation products of arachidonic acid (prostaglandin F2a (PGF2a). In patients with progressive disease, levels of PGF2a were lower in plasma but higher in cerebrospinal fluid (CSF), and increase was correlated with increased disease severity (*p* < 0.044) and patient age (*p* < 0.022) [9]. However, other studies have reported the absence of correlation between oxidation products in CSF and disease activity 7 [10]. The role of oxidative stress as a pathogenetic mechanism in progressive SM is still unclear and controversial.

Then, the development of therapies that effectively treat patients with SPMS and PPMS forms is linked to the better understanding of the disease pathogenesis. The role of microglia activation, chronic oxidative stress, mitochondrial damage in axons, and age-related iron accumulation has to be further investigated. Then, the perfect treatment should have anti-inflammatory, regenerative, and neuroprotective effects [11].

## 3. Is the Prediction of Progressive Disease Course Possible?

The research of biomarkers of disability’s prediction in PMS is frustrating so far. The development of novel biomarkers in MS has so far been limited because several candidates’ lack of specificity, reproducibility, and accessibility [12]. 

In recent years several measures of risk estimation were developed for predicting clinical course in MS, but they are not specific for the PMS forms. The Bayesian Risk Estimate for MS at Onset (BREMSO) has been formulated for giving an individual risk score to convert from RR to SP course with the employment of scoring systems and predictive models, considering clinical and demographic data collected at MS onset [13].

In recent decades, increasing attention was focused on Magnetic Resonance Imaging (MRI) as a tool for providing prognostic information, and to follow the progression in clinical trials and practice. Conventional MRI has become the main tool in the MS follow-up [14]. In clinical practice, abnormal MRI findings are considered the most informative predictors of future disease activity in short-term, mid-term, and long-term studies. Grey matter atrophy occurs early in MS and has been measured in all MS, with PPMS showing greater and earlier spinal cord atrophy than RRMS [14,15,16,17,18,19,20,21]. Such atrophy correlates with clinical scores and appear to be good biomarkers for disability; they have also been demonstrated to be reliable measures for assessing disease progression [22,23]. Cervical cord volume has been shown to decrease significantly over two years in PPMS, demonstrating potential utility within the timeframe of most clinical trials [24]. However, the atrophy measurements were shown to be unspecific, although they are clearly sensitive. MR spectroscopic imaging study on grey and white matter in PPMS showed a reduction in the concentration of total N-acetyl-aspartate, and glutamate-glutamine in PPMS. The Expanded Disability Status Scale Score (EDSS), a scale used for the measurement of disability, correlated with the total N-acetyl-aspartate concentration in cortical grey matter of PMS patients [25].

Conventional MRI has been shown to be useful in terms of prognostic values only in RRMS, and not for PMS [26,27]. Several studies have focused on brain atrophy, showing its relevant clinical impact in the diagnostic phase and in predicting subsequent disability progression both in RR MS and in PP MS [28,29,30]. A recent study published by the MAGNIMS group included 261 MS patients who had MRI at baseline, and after one to two years and EDSS scoring at baseline and after 10 years. In the whole patient group, after correction for imaging protocol, whole brain and central atrophy were good predictors of EDSS at 10 years [31].

A further step forward is the comprehension of the pathological mechanisms related to the accumulation of irreversible disability in MS obtained by the analysis of grey matter atrophy; in detail, some cortical and deep grey matter areas were more prone to inflammatory and degenerative damage, and when damaged, some cortical areas had a greater impact on the accumulation of physical and cognitive disability [32,33,34,35,36,37]. 

In particular, thalamus and cerebellum were consistently related to clinical disability and its progression over time. Thalamus was found to be one of the earliest structures to atrophy in MS subjects, and its atrophy was correlated with changes in EDSS [33]. Moreover, in a longitudinal study, baseline thalamic fraction (odds ratio = 0.62) was identified as predictor of worsening disability at eight years [34]. Cerebellum has been indicated as a preferred site of lesions in PMS. Here, cerebellar cortex was found to be affected in up to 92% of its extension [36,37]. In a recent five-year longitudinal study, cerebellar cortical atrophy, together with age and cortical lesion load, were indicated among the predictive parameters of conversion from RRMS to SPMS [38].

Beyond diffuse GM damage, the relevance of cortical damage in determining disability has been pointed out by the strong correlation observed between focal GM damage, as visible by double inversion recovery (DIR) sequence and clinical progression. Indeed, a high number of cortical lesions has been demonstrated to characterize patients suffering from severe course with severe cortical atrophy and cognitive impairment [39]. In a five-year longitudinal study on more than 300 MS patients with different clinical phenotypes, patients with a high cortical lesion load at baseline showed worse clinical evolution. This was observed in all clinical subsets [40].

In the last 10 years, magnetization transfer and diffusion tensor imaging gave the most interesting results. It was shown that in all MS phenotypes, the baseline grey matter magnetization transfer ratio and average lesion magnetization transfer ratio are independent predictors of disability [41]. In a longitudinal study on 54 PMS patients, lower level of disability and grey matter damage evaluated at study entry on the base of average grey matter mean diffusivity identified patients with a higher risk of disability progression after five years [42]. A recent prospective study showed that the fractional anisotropy of normal appearing gray matter and T2 lesion load were predictors of a severe EDSS score [43]. Diffusion tensor imaging on the spinal cord in MS revealed that baseline cord cross-sectional area and its fractional anisotropy correlated with an increase in disability at any follow-up [44].

All together, these studies confirmed that neurological and neuropsychological disability progression in PMS is poorly evaluated by conventional clinical and radiological predictors. We need to achieve more accurate estimates of useful predictors in PMS, including unconventional and qualitative markers which are not yet currently available or practicable in routine diagnostics.

## 4. Biological Markers in Progressive Multiple Sclerosis (PMS)

The phenotypic expression of MS is highly heterogeneous; in each patient inflammatory, neurodegenerative, and reparative elements manifest different levels of expression [45]. The ideal biomarker should be specific, reliable, and easy to obtain and reproduce.

The evolving development of proteomics led to novel techniques for studying potential MS-biomarkers in biological fluids, especially in the cerebrospinal fluid (CSF). In the PMS, neurofilament proteins and increased levels of antibodies to the neurofilaments light subunit have been found in PP and SPMS CSF showing good correlations with clinical disability and brain atrophy. Neurofilaments, the major axonal cytoskeleton proteins, represent biomarkers for monitoring axonal damage [46,47]. Moreover, antibodies to various gangliosides [48], and other neuronal antigens have been described as being associated with disease progression in PMS patients [49].

The CSF levels of oxidation products are found to be four-fold higher in patients with PPMS but, as previously discussed; other studies have not confirmed the correlation between oxidative stress and worse clinical course [10,50].

Another developing research is about microRNAs (miRNAs). They constitute a recently discovered class of non-coding RNAs and their functions consist in RNA silencing, and in post-transcriptional regulation of gene expression [51]. The profile expression of circulating miRNAs in PPMS and SPMS was assessed and the overexpression of four miRNAs was associated with both disease courses. In detail, the upregulation of miR-191-5p was found in both progressive MS subtypes, while miR-376c-3p was over expressed only in PPMS. In order to validate their specificity for progressive forms of MS, additional studies on a large PMS cohort are needed [52].

## 5. Therapeutic Advances and Future Prospects in Progressive Multiple Sclerosis (PMS): State of the Art

More than 30 phase II or III therapeutic clinical trials, including persons with PMS, have been completed over the last three decades [5]. The only disease modifying drug (DMD) licensed by the EMA and FDA for no relapsing SPMS is Mitoxantrone, but in clinical practice its use is limited because of its cardiotoxic effects and lifetime risk of leukemia [53]. Currently, no EMA and FDA-approved therapies are disposable for the treatment of PPMS [54].

Some therapeutic agents used off-label showed some efficacy in halting disability progression in progressive MS by reducing the inflammatory processes.

### 5.1. Therapeutic Agents under Investigation

#### 5.1.1. Adrenocorticotropic Hormone

Adrenocorticotropic Hormone (ACTH) exerts anti-inflammatory effects and may have neuroprotective effects in spinal cord injury and ischemic brain injury [55,56]. A phase II trial of ACTH in progressive MS is ongoing (NCT01950234).

#### 5.1.2. Biotin

Biotin is a B-vitamin and its high daily dose administration was shown to have an impact on SPMS and PPMS in reducing disease progression [57]. Biotin could increase mithocondrial functions, according to PMS pathogenetic model of a mismatch between increased energy demand by the damaged nerves and decreased energy production because of damage to the mitochondria. The preliminary results of a phase III trial in PMS (NCT02220933) [58].

#### 5.1.3. Amiloride, Fluoxetine, and Riluzole

A phase II trial of Amiloride, Fluoxetine, and Riluzole (compared with placebo) is currently ongoing in PMS (NCT01910259). Amiloride is able to inhibit acid-sensing ion channels, a property that has been linked to neuroprotective effects [59,60]. Riluzole shows an antiglutamatergic profile, and it is the only licensed disease-modifying treatment for amyotrophic lateral sclerosis [61]. Fluoxetine is a selective serotonin reuptake inhibitor that enhances the production of the brain-derived neurotrophic factor in animal models and suppresses microglia activation and NF-κB [62].

#### 5.1.4. Remyelination Agents

Among agents proposed for remyelination in MS, there is a cell surface protein in neurons; it is the so called “Anti-leucine-rich Repeat and Immunoglobulin-like Domain Containing Neurite Outgrowth Inhibitor Receptor-interacting Protein-1 Antibody” (LINGO-1) [61,62,63]. It inhibits the differentiation of oligodendrocyte precursor cells to mature oligodendrocytes. An ongoing phase II trial (BIIB033) is investigating anti-LINGO-1 in patients with RRMS and SPMS (NCT01864148) [64].

#### 5.1.5. Domperidone

Domperidone is a dopamine-2 receptor antagonist and it increases prolactin secretion by suppression of dopamine inhibition. An ongoing phase II trial (NCT02308137) of oral Domperidone in SPMS is based on the supposed role of prolactin as a remyelinating therapy in MS [65].

#### 5.1.6. Erythropoietin

Erythropoietin (EPO), showed neuroprotective abilities in animal models [66]. A small open-label trial of EPO in PPMS showed improvement of motor function with high-dose EPO [67]. A phase II trial of EPO in PMS is planned (NCT01144117). Furthermore, in EAE studies, EPO has been shown to reduce axonal injury and demyelination [68].

#### 5.1.7. Hematopoetic Stem Cell Transplantation

The utilization of hematopoietic cells transplantation has been proposed to reset immune system for advanced form of MS [69,70]. In detail, the open-label trial was reserved to SPMS and contemplated the infusion of autologous bone marrow-derived mesenchymal stem cells. These infusions improved visual acuity and visual evoked response latency [71]. Both SPMS and PPMS are actually also undergoing a phase II trial of autologous bone marrow infusion (NCT01815632).

#### 5.1.8. Ibudilast

Ibudilast reduces levels of tumor necrosis factor-α acting as non-selective phosphodiesterase inhibitor [72,73,74]. An ongoing phase II trial is studying Ibudilast for PPMS (NCT01982942).

#### 5.1.9. Idebenone

Idebenone, is actually studied (phase II ongoing trial NCT01854359) in PPMS for its antioxidant properties because it is a synthetic analog of coenzyme Q10 [75,76].

#### 5.1.10. Lipoic Acid

Lipoic acid has antioxidant properties and it was studied for several diseases associated with oxidative stress. It was also studied in phase II trials for SPMS (NCT01188811) [77].

#### 5.1.11. Lithium

Lithium is one of the oldest antipsychotic medications and it inhibits glycogen synthase kinase-3 (GSK-3), a protein kinase with a role in regulation of inflammatory processes [78]. A pilot phase I/II trial of Lithium in PMS is ongoing (NCT01259388). The trial is based on the results of EAE studies which have shown that pre-treatment with Lithium could suppress the onset of disease activity [79]. Therefore, GSK-3 could become a target treatment for PMS.

#### 5.1.12. Masitinib

Masitinib is a tyrosine kinase inhibitor that modulates degranulation of mast cells [80]. It has been studied in a phase II trial, but with no statistically significant effect on clinical progression in PMS [81]. A phase IIb/III study of Masitinib in patients with relapse-free SPMS or PPMS is in progress (NCT01433497).

#### 5.1.13. MIS416

MIS416 was originally developed as a vaccine adjuvant [82]. MIS416 has been suggested to modulate T-cell-mediated autoimmune responses in animal models [82]. MIS416 was initially used under compassionate use legislation in New Zealand for SPMS. Recently, in a phase Ib/IIa clinical trial, MIS416 was shown to suppress the development of proinflammatory T helper, and to increase the serum levels of IFN-γ and IFN-γ-associated proteins in 19 patients with SPMS [83]. A phase IIb trial is underway in PMS (NCT02228213) [84].

#### 5.1.14. NeuroVax

Vaccine strategies have been pursued in the prospection of more personalized therapies for MS [85,86]. A phase II study of NeuroVax (a TCR peptide vaccine; Immune Response BioPharma, Atlantic City, NJ, USA) in patients with SPMS is planned to start soon (NCT02057159).

#### 5.1.15. Oxcarbazepine

In experimental animal models, the partial blockade of sodium channels showed neuroprotective properties [87,88,89].

Oxcarbazepine was studied in a phase II trial which is ongoing (NCT02104661); it has the aim to assess the change in content of neurofilament light chain in CSF, considered a neurodegeneration marker [90].

#### 5.1.16. Simvastatin

Statins (hydroxymethylglutaryl-CoA reductase inhibitors) have been studied for their immunomodulatory and neuroprotective effects that they probably exercise through the improvement of cerebrovascular hemodynamics [91]. Moreover, Simvastatin seems to significantly reduce brain atrophy when compared with placebo, as showed recently a II phase trial [92].

#### 5.1.17. Siponimod

Siponimod (BAF312) is an oral selective sphingosine-1-phosphate (S1P) receptor modulator acting on subunits: SIP-1and SIP-5 [93,94,95,96]. Recently, at the 32th European Committee for Treatment and Research in Multiple Sclerosis (ECTRIMS 2016), the results of the phase III EXPAND study were presented. The primary efficacy outcome was three months of Confirmed Disability Progression (CDP) measured by EDSS, and the study meets its primary end point of reducing the risk of CDP by three months [96].

#### 5.1.18. Sunphenon Epigallocatechin-3-gallate

Sunphenon epigallocatechin-3-gallate (EGCg) is a polyphenolic flavonoid extracted from green tea leaves.

It has beneficial effects ranging from anti-tumor, antioxidant, and anti-inflammatory [97,98]. It is actually under investigation for PMS in a phase II/III trial study (NCT00799890).

### 5.2. Monoclonal Antibodies

#### 5.2.1. Natalizumab

The two monoclonal antibodies actually employed for the RRMS therapy are Natalizumab and Alemtuzumab and several trials are investigating them [99].

Natalizumab is a humanized monoclonal antibody that binds to the α4-subunit of α4β1 and α4β7 integrins, expressed on the surface of lymphocytes and inhibits the α4-mediated adhesion of their counter-receptor(s).

These molecular interactions prevent transmigration of lymphocytes across the blood–brain barrier (BBB) into o inflamed parenchymal tissues [100]. In an open-label phase IIa trial, Natalizumab has been tested in patients with PPMS, showing the ability to reduce CSF inflammatory, axonal, and demyelination biomarkers. However, preliminary results from a phase III trial of Natalizumab in patients with SPMS (ASCEND; NCT01416181) showed no efficacy in terms of delaying disability assessed by EDSS, Timed 25-Foot Walk, and 9-Hole Peg Test [101].

#### 5.2.2. Anti-CD20 Monoclonal Antibodies

Anti-CD20 monoclonal antibodies are Rituximab and Ocrelizumab.

Rituximab is a chimeric IgG1 monoclonal antibody while Ocrelizumab is humanized [100]. In a phase III trial enrolling PPMS, Rituximab did not delay the disability progression compared to placebo. The results from subgroup analyses showed a slight beneficial effect in younger patients (aged < 51 years), particularly those with inflammatory lesions on brain MRI [101]. A phase II trial of combined IV and intrathecally administered Rituximab (vs. placebo) in patients with SPMS is currently ongoing (NCT01212094).

A phase III trial of Ocrelizumab in patients with PPMS (ORATORIO) was designed (NCT01194570) [102]. Patients were required to have an elevated IgG index, two or more oligoclonal bands, an EDSS of 3.0 to 6.5, and an age of 18 to 55 years. PPMS on Ocrelizumab arm showed a risk reduction of reaching the primary endpoint (time to onset of 12-week confirmed disability progression) by 24% than placebo (*p* = 0.0321).

## 6. Future Research Directions and Conclusions

There is ongoing growing interest in new therapies for PMS and this provides reasons for being optimistic for the future.

However, we have collected frustrating results so far and this is partly related to the lack of knowledge about PMS pathogenesis; and the model of clinical trial design that could not be relevant to PMS. Our efforts have, so far, been addressed to modulating the inflammatory activities, but PMS forms are characterized by neurodegeneration which is the consequence of variable levels of myelin loss and axonal damage. The potential effectiveness of pharmacological agents for MS is based on EAE models, but it is an inflammatory model. Ideally, clinical trials should define the inclusion criteria of the enrolled population, given an extreme heterogeneity of findings. The results based on subgroups should not be post hoc exploratory analysis. Moreover, to design a better personalized approach to PMS treatment, we first understand which clinically meaningful outcomes are for persons with PMS. For instance, our concept of disability is based mainly on physical (particularly on deambulation) disability.

The actual disability outcome measures are not adequately validated for PMS because often the patient-perceived health status or quality of life is not considered. The EDSS is based on the standard neurological examination, which is inherently subjective; and how EDSS scores are calculated raises various issues. Scores from 4.0 to 7.5 are based primarily on the distance the patient can walk and does not account for other forms of disability (hand function, cognitive impairment, etc.). The patient-reported outcomes (PROMs) are based on reports made directly by the patients, without any interpretation by a clinician or anyone else. The European Medicines Agency is encouraging the use of PROMs, but has not provided specific regulatory guidance. PROMs in MS include general instruments, such as the medical outcomes study short form-36 (assesses vitality, physical functioning, pain, general perceptions of health, physical, emotional and social functioning, and mental health), MS quality of life-54 (derived from short form-36), MS quality of life inventory, and MS impact scale-29. These multidimensional scales measure several domains, but PROMs can focus on single domains, such as ambulation (MS walking scale-12), depression (Beck depression inventory and patient health questionnaire), or fatigue (modified fatigue impact scale). No specific scales were developed for PMS.

An important challenge is the optimization of rational approaches for the identification of candidate biomarkers [103]. Then, new studied biomarkers should capture the neurodegenerative aspects of the disease.

A personalized approach in PMS should define a personal prognostic profile. To do that, we would look for every patient at both clinical activity and MRI disease activity and also the prognostic profile. The problem is that we do not have common guidelines which can be applied with broad brushstrokes across many different patient types.

Unfortunately, we have not been able to figure out who is and who is not going to be a responsive to a certain drug in PMS. We have not been able to determine whether one patient with PMS is going to respond well to the treatment while another patient will not. However, what we have been able to do is to tailor the choice of treatments to take into account the risks that certain patients have for complications from those treatments. By recognizing what the different risks are and how different patients have a higher or lower predisposition to those risks, we can better tailor and personalize the treatment. The monoclonal antibodies, and in particular Natalizumab, raised the interest on opportunistic infections, such as progressive multifocal leukoencephalopathy (PML). Are we exposing our PMS patients to unnecessary risk? Before we even start with any treatment, we need to have some idea of what the patient’s course has been and what the risk is for new progression. Many trials conducted to evaluate treatments for PMS did not select subsets of patients with similar disease characteristics, so investigators have to be encouraged to test treatments based on biomarkers that could select potential responders.

We have learned, over the years, the clinical factors that might tell us whether a person with PMS does or does not have a better prognosis. Good prognostic indicators include younger age at onset, being female, being white, and the nature of the first attack. In the last years, the use of MRI as prognostic indicator increased dramatically. We can look at the MRI and see how many lesions, or how much of the brain is involved with MS. We can also assess if the spinal cord is involved and how many lesions are there. We can look at the severity of the lesions by calculating how many of them are T1 black holes, which are a more destructive type of lesion than are the typical T2 lesions. We can also look at the degree of atrophy visible on the MRI. Then, neuroradiological support could help the clinician to stratify patients with PMS and to predict disease activity or progression; moving towards a personalized treatment approach.

This information can help us to choose the appropriate therapeutic weapon.

We can look to the future for important new developments. It is an exciting time; PMS is certainly one of the fastest-moving conditions in the neurology field.
